# BUILDING THE COLLABORATIVE COMMUNITY-ACADEMIC TEAM, DESIGNING OBJECTIVES, AND GETTING FUNDED

**DOI:** 10.1093/geroni/igad104.0029

**Published:** 2023-12-21

**Authors:** Zachary Baker, Sheryl Fairbanks, Warren Wolfe, Robbin Frazier, Ashley Millenbah

**Affiliations:** Arizona State University, Tempe, Arizona, United States; University of Minnesota, Minneapolis, Minnesota, United States; Freelance Journalist, Roseville, Minnesota, United States; University of Minnesota, Center for Healthy Aging and Innovation, Minneapolis, Minnesota, United States; University of Minnesota, Minneapolis, Minnesota, United States

## Abstract

Having attended the Former Dementia Caregiver Re-Entry Initiative since 2019, its benefits were readily apparent but two limitations were identified: 1) two facilitators (i.e., Wolfe and Fairbanks) cannot serve the millions of Bereaved Dementia Caregivers in the US alone and 2) members have primarily been non-Hispanic White. These realizations inspired efforts to bring the group to more Bereaved Dementia Caregivers. The present community-created model of helping Bereaved Dementia Caregivers deserved community-integrated leadership if that model was to be effectively adapted and disseminated. Our team’s ambition is to bring the group to racially and ethnically minoritized Bereaved Dementia Caregivers, broadly. However, we felt that we could most effectively do this in stages. Rather than trying to figure out how to make the model fit for all people, we homed in on African Americans, with whom we had many local ties. We also took a step back (at the behest of Ms. Frazier) to not only ask about how to adapt the model for a need we assumed existed, but also to explore the strengths of African American communities and ask if unmet needs existed. This model had the benefits of intellectual humility, respect for the persons we were working with, and strengthened the value of our funded research (i.e., even if we found there weren’t unmet needs, we would be able to learn of strengths that we might be able to share with other communities who do have unmet needs).

